# Pre-school Teachers' Stereotypes and Self-Efficacy are Linked to Perceptions of Behavior Problems in Newly Arrived Refugee Children

**DOI:** 10.3389/fpsyt.2020.574412

**Published:** 2021-01-27

**Authors:** Sandy Chwastek, Birgit Leyendecker, Anna Heithausen, Cristina Ballero Reque, Julian Busch

**Affiliations:** Faculty of Psychology, Child and Family Research, Ruhr-University Bochum, Bochum, Germany

**Keywords:** mental health, refugee, pre-school, teacher, perception, SDQ, early education, childcare

## Abstract

**Introduction:** Since 2015, increased numbers of refugee families with pre-school-aged children have arrived in Germany. In pre-schools, teachers' professional competence for teaching those children and adapting to their socio-emotional needs has become increasingly important. Previous research linked teachers' stereotypes and cultural beliefs to their self-efficacy and enthusiasm when teaching immigrant children. This study investigated the links between domains of pre-school teachers' professional competence (i.e., negative stereotypes, multicultural beliefs, self-efficacy, and enthusiasm when teaching newly arrived refugee children), and examined whether teachers' professional competence was linked to their perceptions of newly arrived refugee children's behavior problems.

**Method:** In a cross-sectional self-report survey, *N* = 147 German pre-school teachers reported on their professional competence and completed the Strengths and Difficulties Questionnaire (SDQ) for a selected refugee child from their pre-school group. We used regression modeling to link teachers' negative stereotypes and multicultural beliefs to their self-efficacy and enthusiasm for teaching refugee children. Next, we examined the links between teachers' beliefs, values, and motivational orientations to their ratings on the SDQ subscales. Last, we linked demographic data on teachers and children to teachers' professional competence and SDQ ratings.

**Results:** Teachers with more negative stereotypes toward newly arrived refugee children and less agreement with multicultural beliefs reported lower self-efficacy and enthusiasm for teaching newly arrived refugee children. Teachers with more negative stereotypes perceived more hyperactivity/inattention and total difficulties. Teachers with higher self-efficacy perceived less hyperactivity/inattention, less total difficulties, and more prosocial behavior. Additionally, teachers who had more experience with refugee children reported more negative stereotypes and higher agreement with multicultural beliefs. Teachers having more overall work experience perceived more total difficulties. Boys were perceived to display more externalizing behavior problems, less prosocial behavior, and more total difficulties. Older children were perceived as displaying more prosocial behavior and children from African countries were perceived as displaying more conduct problems.

**Discussion:** Our findings suggest that pre-school teachers' stereotypes and self-efficacy might be related to perception biases concerning newly arrived refugee children's externalizing behavior problems. Implications for the professional development of pre-school teachers and teacher-informant diagnostics of refugee children's socio-emotional needs are discussed.

## Introduction

Since 2015, large numbers of forcibly displaced children under the age of six have been arriving in Germany from conflicted and deprived regions [46.316 in 2019; (1)]. Increased levels of behavior problems of refugee children have been found in several countries, including evidence on the early childhood period (2–5). For example, elevated levels of aggressive behaviors, inattention, and peer problems were also found in refugee children attending early education programs in Germany when compared to norm data (6). Refugee children are exposed to large numbers of risk factors throughout migration periods. Many of those risk factors render the children extremely sensitive to the quality of child care services (7–9). Early childhood teachers' accurate perceptions and handling of refugee children's socio-emotional needs can promote adjustment processes and positive development during resettlement periods.

### Early Education and Challenges for Teachers

In Germany, the great majority of all children aged three to six attend pre-schools, which are required to provide the learning basis for transitioning into primary school at around age 6 (10). They are in a unique position to support children at risk, as they can consider their specific needs without having to focus on strict academic content. Prior, early education was found to reduce the impact of risk factors on child development and school performance in low-income children (11, 12). Due to their backgrounds and experiences, such as interruption of formal education, young refugee children require teachers to be more flexible and to consider their specific needs (13). However, teachers often lack experience or training for working with refugee children. Teachers reported feeling insecure in effectively detecting and serving refugee children's specific needs (14–16) and to find overcoming language barriers as well as communication with the parents difficult (17). Enhancing teachers' abilities to detect specific needs and behavior problems can improve the positive impact of early education on refugee children. Especially when identifying socio-emotional needs in refugee children, understanding behaviors, overcoming stigma, cultural and linguistic barriers, engaging with parents, and having a Western understanding of mental health were identified as challenging for teachers (18). Here, teachers' professional competence seems to play an important role. To the best of our knowledge, no study has yet addressed the relationship between pre-school teachers' professional competence and their perceptions of behavior problems in refugee children.

### Definition of Constructs

In their model of professional competence, Hachfeld et al. (19) define teachers' professional competence as an important component of effective teaching processes in multicultural settings. Teachers' beliefs and values (comprising stereotypes and cultural beliefs) and teachers' motivational orientations (comprising self-efficacy and enthusiasm for teaching immigrant students), amongst others, were identified as central domains influencing the teaching of culturally diverse children. In this study, we selected our constructs based on this model. We focus on multicultural beliefs, which are, besides colorblind beliefs, an aspect of cultural beliefs. Teachers with a higher agreement with multicultural beliefs recognize that students from different socio-cultural backgrounds have different perspectives and beliefs, value their students' different backgrounds as enriching and are open to embracing them in their teaching (20). This is opposed to colorblind beliefs. Teachers with colorblind beliefs believe that students' different cultural backgrounds should not be taken into account while teaching and teachers should focus on students' similarities (20). Multicultural beliefs were linked to more reported willingness to adapt one's teaching to immigrant students (19) such as ensuring that all students, despite their different cultural or linguistic backgrounds, are able to follow the lessons.

Hachfeld et al. (19) define stereotypes as negative expectancies toward immigrant students' educational engagement and motivations. We narrow down the definition to the extent to which the teachers perceive newly arrived students' refugee backgrounds as a burden for teaching. Self-efficacy and enthusiasm are looked at specifically in the context of teaching immigrant students (19). Following the model, self-efficacy is defined as the teachers' beliefs in their capabilities to successfully organize and execute specific teaching tasks in a particular context (21). We define teachers' enthusiasm as the degree of positive experiences during teaching (22) and their positive attitude to work with culturally diverse children. Differently to Hachfeld et al. (19), we relate teachers' professional competence explicitly to newly arrived refugee children in pre-school context instead of immigrant students in general.

### Prior Research on the Relations of Teacher's Professional Competence

Multicultural beliefs were related to higher levels of self-efficacy and enthusiasm for teaching immigrant students as well as less negative stereotypes when teaching them (19, 23), and were negatively related to prejudicial thoughts (20). Expanding those findings, Bangura (24) found cultural awareness to positively predict multicultural self-efficacy of teachers in training. Similar to multicultural beliefs, perceiving immigrant students as an asset instead of viewing them negatively was related to higher immigration-related self-efficacy in primary and secondary school teachers (25). Although large numbers of pre-school-aged refugee children have arrived in Germany, Hachfeld's et al. (19) model has not yet been applied to investigate adaptations of early education to refugee children's specific needs.

### Prior Research on Professional Competence and the Perception of Students

Evidence suggests that teachers' professional competence influences their perceptions of children's behavior problems in educational settings. Different kinds of teachers' beliefs such as those concerning student skills and performances were linked to teachers' perceptions, judgments, and behaviors in the classroom (26). We assume that especially stereotypes and multicultural beliefs are linked to teachers' perceptions of behavior problems in refugee children. The term refugee has the potential to trigger bias in teachers, depending on their stereotypes toward traits associated with this term and their multicultural beliefs. Supporting this notion, students' ethnicity and skin color were linked to teacher ratings and expectations toward students' behavior as well as the feedback teachers provided during instructions (27). Teachers also attributed lower achievement scores to children who had ethnic first names (28). While distinct evidence for pre-school-aged children is limited, similar results were found for assessments of students' performance in primary schools (29). Evidence suggests that teachers report more behavior problems for certain subgroups of immigrant or minority children (30, 31). According to these findings, more stereotypes and less agreement with multicultural beliefs should lead to focusing on children's difficulties instead of their strengths and in consequence to increased perceptions of behavior problems. In contrast, high self-efficacy and enthusiasm could have opposite effects. Along these lines, studies found teachers' self-efficacy beliefs to be associated with their sense of efficacy in resolving cultural conflicts (32). Additionally, Gibbs (33) discussed that teachers' sense of control influences how they think, feel, and teach. Thus, higher self-efficacy could be related to lower teacher's perceptions of behavior problems in refugee children.

### The Association of Teachers' Perceptions and Student Outcomes

Assessing teachers' perspectives on students in early education is crucial, as teachers' expectation biases were found to have effects on long-term student performance and also to partly mediate the effects of student characteristics on students' performance (34). Understanding the behavior problems of students increases the odds of teachers' readiness to help them (35). Furthermore, perception biases can put culturally and linguistically diverse children at a disadvantage due to misperceptions of their true learning needs. Brown (36) found teachers to misperceive foreign child behaviors as disabilities, leading to erroneous placements in language disability interventions.

Teachers' professional competence was also directly linked to motivation and achievements in students. In a systematic review of 34 articles on learning problems in refugee children, Graham et al. (37) found teacher stereotyping and low expectations to be two of the major risk factors for their students' learning problems. Similarly, teachers' self-efficacy was linked to teachers' behavior, such as trying out new techniques and being more persistent while helping, to students' academic outcomes (38), and it positively influences students' motivation and academic achievements (39). Teachers' enthusiasm was found to predict students' interests (40) and, through instructional quality, students' outcomes (41). According to these findings, accurate pre-school teachers' perceptions, along with their appropriate behavior and guidance, could help refugee children to build strong learning foundations for the transition into primary school.

### This Study

To date, the links between teachers' attitudes and views on the behavior of newly arrived refugee children within early education settings are understudied. However, teachers are regular informants on children's behavior problems and thus important to understand newly arrived refugee children's socio-emotional needs. Therefore, we need to better understand (A) pre-school teachers' professional competence when teaching refugee children and (B) how teachers' professional competence facilitates serving the specific socio-emotional needs of those children. For this purpose, the present study investigates links between teachers' stereotypes, multicultural beliefs, self-efficacy, enthusiasm, and their perceptions of behavior problems in young refugee children. We hypothesize that (H1) teachers with less negative stereotypes and higher agreement with multicultural beliefs toward refugee children are likely to display increased levels of self-efficacy and enthusiasm when teaching newly arrived refugee children; (H2) teachers with more negative stereotypes and less agreement with multicultural beliefs are likely to perceive more behavior problems in newly arrived refugee children; (H3) teachers with higher self-efficacy and enthusiasm are likely to perceive fewer behavior problems in newly arrived refugee children. Additionally, we link the most important demographic child and teacher data to teachers' competence and ratings on behavior problems. This study provides important evidence on the teacher-related determinants of responding to the mental health needs of newly arrived refugee children in early education services. Implications for the professional development of pre-school teachers and their work with refugee children are discussed.

## Materials and Methods

### Procedure

We surveyed teachers who worked in pre-school facilities in the German federal state of North Rhine-Westphalia (NRW) online between May and July 2018. NRW received more than **one** quarter of all refugees in Germany. The survey period corresponds with the last quarter of the German academic year in pre-schools. First, we advertised our online survey to teachers in pre-schools via telephone, and later additionally via e-mail to increase the number of participants. When contacting pre-schools, we made sure to include both rural and urban areas. This sampling strategy intended to diversify our study sample increasing the generalizability of our results. Later, we additionally contacted pre-school teachers via a few selected social media channels, while clearly stating our inclusion criteria. Preschool teachers were included if they worked in pre-schools within NRW with at least one refugee child in their group who arrived after the year 2014. In each pre-school group, only one teacher was asked to participate. This ensured that children were not rated twice and children and groups were independent within our sample. Teachers filled in an online survey using the platform “Qualtrics” (SAP). They were asked to report on their professional competence and on the behavior problems of one selected refugee child of their group. Teachers were asked to select the child that had been attending their pre-school group for the longest period. As an incentive for participation, two tablets were raffled among the participants. The study protocol was approved by the Ethics Committee of the Faculty of Psychology of the Ruhr-University Bochum.

### Participants

Overall, *N*_*total*_ = 240 teachers accessed the survey. We filtered out flawed datasets based on missing data analysis and, in preparation for statistical modeling, visual inspection of residual plots. We only considered fully completed questionnaires for further analysis. *N* = 11 teachers left the survey after filling in their demographic data and *n* = 82 teachers after filling in the scales of teachers' competence. We assume that those participants were not interested to fully complete the survey for unknown and random reasons. The effective sample consisted of *N* = 147 early childhood teachers. Table 1 shows descriptive information on the participating teachers and the pre-schools they work in. Notably, more than two-thirds of teachers reported having “rather a lot” or “a lot” of experience working with refugee children. The most common languages spoken by newly arrived children in the pre-school facilities were Arabic (in 82.19% of all participating pre-schools), Kurdish (44.52%), Albanian (39.73%), Farsi (29.45%), and Serbian (21.23%). All children in this study learned German as their second language. The regional distribution of the facilities was nearly equally spread, with 46.94% being in rural or rather rural and 53.06% being in urban or rather urban areas. Table 2 provides demographic information on the assessed refugee children. The most common countries of origin were Syria (28.35%), Iraq (10.24%), and Afghanistan (7.09%).

**Table 1:** Descriptives on pre-school teachers and facilities.

	**M**	**SD**	**Mdn**	**Min**	**Max**	
Age (*n* = 142)	41.12	11.88	42.5	19	63	
Work experience in months (*n* = 145)	229.22	142.75	240	9	528	
Gender (*n* = 147)	Male					4.76%
	Female					95.24%
School-leaving qualification (*n* = 147)	Secondary school certificate					36.73%
	Vocational diploma					43.54%
	A-Levels					19.73%
Qualification (*n* = 146)	Educator					78.08%
	Employee with social and curative school qualification					8.22%
	Other					13.69%
Role at the facility (*n* = 147)	Pre-school educator					37.41%
	Pre-school manager					53.74%
	Other					8.84%
Work experience with refugee children (*n* = 144)	No experience					0.69%
	Rather no experience					29.17%
	Rather a lot of experience					52.78%
	A lot of experience					17.36%
Own migrant background^a^ (*n* = 147)	Yes					10.88%
	No					89.12%
	**M**	**SD**	**Mdn**	**Min**	**Max**	
Total number of children in facility (*n* = 146)	74.36	39.03	67	10	369	
Newly arrived refugee children^b^ (*n* = 137)	18.09	10.18	19	1	36	
Current number of refugee children (*n* = 142)	14.16	9.54	13.5	1	31	

*n, number of cases; M, mean; SD, standard deviation; Mdn, median; Min, minimum stated by participants; Max, maximum stated by participants*.

a *Either one parent or the teachers themselves migrated to Germany*.

b *Newly arrived refugee children within the last 4 years*.

**Table 2:** Descriptives on refugee children.

	**M**	**SD**	**Mdn**	**Min**	**Max**	
Age in months (*n* = 145)	60	14.47	60	15	83	
Time in pre-school in months (*n* = 145)	20.75	11.3	20	2	66	
Time in Germany in months (*n* = 104)	32.8	14.69	36	2	65	
						**Rel number**
Gender (*n* = 146)	Female					50.68%
	Male					49.32%
Region of origin (*n* = 127)	Southeastern Europe					12.59%
	Africa					10.23%
	Western Asia					47.25%
	South and Eastern Asia					11.02%
	Other					18.90%

*n, number of cases; M, mean; SD, standard deviation; Mdn, median; Min, minimum stated by participants; Max, maximum stated by participants. Southeastern Europe = Albania, Greece, Montenegro, Romania, Serbia. Africa = Egypt, Eritrea, Morocco, Nigeria. Western Asia = Iran, Iraq, Syria, Turkey. South and Eastern Asia = China, Pakistan, Afghanistan*.

### Measurement

Teachers completed a set of standardized self-report questionnaires. First, teachers reported on their personal backgrounds and the pre-school facilities. Second, following the conceptual propositions by Hachfeld et al. (23) and Hachfeld et al. (19), teachers reported on their stereotypes and multicultural beliefs as central domains of their beliefs and values, and on their self-efficacy and enthusiasm for teaching refugee children as central indicators for their motivational orientations. Third, teachers reported on behavior problems of a systematically selected child using a standardized scale. For controlling order effects, scale items were presented randomly per construct. Mean values were computed for each scale of professional competence.

#### Demographic Information on the Teachers and Pre-school Facilities

We obtained socio-demographic information on the teachers (gender, age, professional qualification, position within the facility, experiences in educational work in general and with refugee children, migration background) and information on newly arrived children in the pre-school facilities (number of children within the facility, number of refugee children within the facility within the last four years, current number of refugee children within the facility, languages spoken by the refugee children, location of the facility). In addition, teachers provided information on the socio-demographic backgrounds of the selected children (gender, age, time spent within the facility, time spent in Germany, country of origin).

#### Teachers' Stereotypes and Multicultural Beliefs

In our study, stereotypes toward refugee children reflect the extent to which the teachers perceived newly arrived children's refugee backgrounds as a burden for teaching. We selected items following the SIMCUR survey (unpublished) that measured teachers' stereotypes toward immigrant children in primary schools and adapted wording to early education contexts (i.e., exchanging words such as school and pre-school). Items for the SIMCUR survey were constructed following the multicultural beliefs and stereotype scales from Hachfeld et al. (23). Whereas, the latter focused on students‘ school-related motivation and effort, items in the SIMCUR survey were framed to address newly arrived children's challenges in pre-school such as coping with the new surroundings and needing additional support. We assessed stereotypes using a 4-point Likert scale (1 = not accurate, 4 = very accurate).

According to Hachfeld et al. (20), multicultural beliefs measure how strongly teachers endorse that cultural backgrounds of refugee children should be considered in teaching. We selected four items of the Teacher's Cultural Beliefs Scale [TCBS; (20)] and adapted wording to early education contexts. In line with the TCBS, we used a 6-point Likert scale (1 = not accurate, 6 = very accurate). Hachfeld et al. (23) reported both scales to have a good internal consistency with α = 0.75 for multicultural beliefs and α = 0.88 for stereotypes. Items of both scales can be seen in Table 3.

**Table 3:** Items assessing stereotypes and multicultural beliefs.

Stereotypes
1. In general, children from newly arrived families need more support. 2. In general, children from newly arrived families are an additional burden for the pre-school teacher. 3. Children from newly arrived families have more difficulties to cope with daily routines in pre-school.
Multicultural beliefs
1. During the apprenticeship, pre-school teachers should learn how to cope with cultural diversity. 2. During conversations with parents of different cultural backgrounds, I try to show consideration for their cultural background. 3. It is important for children to learn that people from other cultures can have different values. 4. In the pre-school classroom, it is important to be responsive to differences between cultures.

*The table represents a translation of the original items*.

#### Teachers' Self-Efficacy and Enthusiasm

We followed the conceptual propositions by Hachfeld et al. (19) to assess teachers' motivational orientations by measuring their perceived self-efficacy and enthusiasm for teaching refugee children. In our study, teachers' self-efficacy reflects their beliefs in their capabilities to successfully organize and execute specific teaching tasks in a refugee context. For both constructs, we used the same items and item numbers as Hachfeld et al. (23), except for changing the wording from “students with a migration background” to “newly arrived children.” Self-efficacy was assessed by four items (e.g., “I am capable of addressing the specific needs of newly arrived children”).

In our study, enthusiasm for teaching refugee students reflects the degree of positive experiences during teaching. It was assessed by the two items “I enjoy caring for newly arrived children” and “I enjoy working with children from different backgrounds.” Participants responded to the items of motivational orientations on a four-point Likert scale (1 = not accurate, 4 = very accurate). Hachfeld et al. (19) reported both scales to have a good internal consistency with α = 0.81 for self-efficacy and α = 0.90 for enthusiasm.

#### Children's Behavior Problems

To assess perceptions of the refugee children's behavior problems, we used the German adaptation of the teacher-report Strengths and Difficulties Questionnaire [SDQ; (42, 43)] for children aged 4 to 17. The SDQ is a 25-item behavioral screening tool, consisting of five subscales (emotional symptoms, conduct problems, hyperactivity/inattention, peer relationship problems, and prosocial behavior). Each subscale consists of five items on a 3-point Likert scale ranging from 0 = “absolutely not true” to 2 = “absolutely true.” Sum scores were computed for each SDQ subscale. Additionally, all scale scores except for prosocial behavior are summed up into a total difficulties score. In Western and Non-Western cultures, the SDQ has demonstrated good cross-cultural validity (44). For scoring and missing data detection, we used a script provided by the authors of the SDQ measure (sdqinfo.org). We thus erased subscales for individual children if more than three items per subscale were missing and only calculated the total difficulties score if no more than two items per subscale were missing.

### Statistical Analysis

All analyses were conducted in R version 1.2.1335. For the first hypothesis, we used multiple linear regression modeling to link teachers' stereotypes and multicultural beliefs to their self-efficacy and enthusiasm in separate models. For the second and third hypotheses, we used multiple linear regression modeling to, respectively, link teachers' SDQ ratings per subscale to their professional competence in separate models. Using the *plot* function for linear models, we visually inspected leverage plotted against standardized residuals and removed *n* = 3 influential data points from the dataset. We then used the function gvlma of the package “*Global Validation of Linear Model Assumptions*” [*gvlma*; (45)] to examine assumptions for linear modeling. The procedure provides information on linearity, kurtosis and skewness of residuals, homoscedasticity, and scaling of the variables. Overall, the assumptions were acceptable across all models. We used the function *vif* of the *car* package for assessing multicollinearity. The variance inflation factor did not suggest multicollinearity and we used linear regression for modeling. As theory substantiated directions for our hypotheses, we used one-sided *p*-values (α = 0.05) for interpreting all regression models. We used Bonferroni–Holm adjustments to control for α-error inflation due to multiple testing (46). For regressing on the SDQ subscales, Bonferroni-Holm correction was used on all four subscales in one step as well as separately on total difficulties scores and prosocial behavior, as those were not part of the proposed hypotheses.

Additionally, we investigated the influence of teacher and child demographics. We conducted multiple linear regression analyses of teacher demographics (work experience, work experience with refugees, school-leaving qualification) on teachers' professional competence (enthusiasm, self-efficacy, stereotypes, multicultural beliefs) and the SDQ subscales. Additionally, we conducted multiple linear regression analyses of child demographics (age, gender, time spent in the pre-school facility, and country of origin) on SDQ subscales, as teachers reported on their professional competences before knowing which child they had to report on. Again, we used the function *gvlma* of the package *gvlma* and Bonferroni-Holm adjustments to control for α-error inflation due to multiple testing.

Based on literature, correlations, and content-wise decisions, we chose three important teacher and four important child variables for additional regression analyses on confounders. We excluded other confounding variables because some were closely related by content and our study sample did not allow for the inclusion of a large number of predictors. The excluded variables were the age of the teachers (as it highly correlated with work experience; *r* = 0.932 *p* < 0.001), qualification of pre-school teachers (as the majority of the teachers', 78.08%, had the required pre-school exam and qualification is highly correlated with teachers' school-leaving qualification), teachers' role in the facilities (as it turned out that teachers who do administrative work were also involved in regular teaching; teachers' migration backgrounds (as the percentage of teachers with a migration background was 10.88%); gender (as the proportion of men in our sample was too small for group comparisons, 4.76%). Furthermore, we excluded the time children had spent in Germany because this variable was only completed for *n* = 104 children.

## Results

In Table 4, we report descriptive information on teachers' stereotypes, multicultural beliefs, self-efficacy, enthusiasm, and perceptions of children's behavior problems. Eight teachers had one missing value in one of the subscales on professional competence. Those teachers were excluded from the analysis of the respective subscale. See Table 4 for reliabilities of all scales. Regarding the measure for children's behavior problems, *n* = 26 SDQ subscales for *n* = 20 children were incomplete (equals 1.23% of all SDQ items).

**Table 4:** Teachers' professional competence for teaching refugee children and measures of children's behavior problems (SDQ).

			**M**	**SD**	**Mdn**	**Min**	**Max**	**Std alpha**
Beliefs and values		Multicultural beliefs^a^ (*n =* 146)	5.23	0.69	5.25	1.5	6	0.66
		Stereotypes^b^ (*n* = 146)	2.92	0.66	3	1	4	0.67
Motivational orientation		Self-efficacy^b^ (*n* = 143)	3.26	0.52	3.25	1.5	4	0.76
		Enthusiasm^b^ (*n* = 145)	3.57	0.59	4	1	4	0.77^†^
SDQ	externalizing	Hyperactivity/inattention^c^ (*n* = 147)	4.15	2.87	4	0	10	0.81
		Conduct problems^c^ (*n* = 147)	2.24	2.45	1	0	10	0.72
	internalizing	Emotional symptoms^c^ (*n* = 146)	2.59	2.39	2	0	10	0.79
		Peer relationship problems^c^ (*n* = 147)	2.82	2.28	2	0	9	0.63
		Prosocial behavior^c^ (*n* = 147)	6.03	2.59	6	0	10	0.78
		Total difficulties score (*n* = 146)	11.77	7.44	10.5	0	33	0.86

*n, number of cases; M, mean; SD, standard deviation; Mdn, median; Min, minimum stated by participants; Max, maximum stated by participants; Std alpha, standardized Cronbach's alpha*.

a 6-point Likert-scale, 1 = “not accurate” to 6 = “very accurate.”

b 4-point Likert-scale from 1 = “not accurate” to 4 = “very accurate.”

c 3-point Likert-scale from 0 = “absolutely not true” to 2 = “absolutely true.”

*For professional competence subscales, mean scores were calculated. For SDQ subscales, sum scores were calculated*.

† *For the subscale “enthusiasm” (two items) the correlation was calculated*.

In the first hypothesis, we expected stereotypes to be negatively associated with self-efficacy and enthusiasm, and multicultural beliefs to be positively associated with self-efficacy and enthusiasm. Our findings support this hypothesis (see Table 5).

**Table 5:** Multiple linear regression modeling for teachers' beliefs and values on teachers' motivational orientations.

**Outcome**	**Predictor**	***b***	***b (95% CI)***	**ß**	***p^a^***	**Fit**
Self-Efficacy	(Intercept)	2.5801	[1.84, 3.32]	−0.0057	0.944	
	Multicultural beliefs	0.2190	[0.09, 0.35]	0.2428	0.004^**^	
	Stereotypes	−0.1563	[−0.28, −0.03]	−0.1979	0.018^**^	Adj. *R^2^*=0.0787^**^
Enthusiasm	(Intercept)	3.0725	[2.32, 3.83]	−0.0176	0.825	
	Multicultural beliefs	0.2231	[0.09, 0.35]	0.2411	0.003^**^	
	Stereotypes	−0.2207	[−0.34, −0.10]	−0.2726	< 0.001^**^	Adj. *R^2^*=0.1173 ^***^

*b, unstandardized regression weights; b (95% CI), 95% confidence intervals for the unstandardized regression weights; ß, standardized regression weights; p, two-sided p-values; Adj. R^2^, adjusted R^2^*.

** *p < 0.01*,

*** p < 0.001

a *Markings indicating significances following one-sided Bonferroni–Holm correction*.

Results on the second and third hypotheses are shown in Table 6. In the second hypothesis, we expected more stereotypes and less agreement with multicultural beliefs to be associated with the perception of more behavior problems. After Bonferroni–Holm correction, we found stereotypes to be positively associated with the perception of hyperactivity/inattention, but not with conduct, emotional or peer problems. Similarly, stereotypes were positively associated with the SDQ total difficulties score. Regression models linking multicultural beliefs to the SDQ subscales yielded no significant effects. Thus, the results only partially support the second hypothesis. In the third hypothesis, we expected higher self-efficacy and enthusiasm to be associated with the perception of fewer behavior problems. After Bonferroni-Holm correction, we only found self-efficacy to be negatively associated with the perception of hyperactivity/inattention, but no other subscale. Additionally, self-efficacy was positively associated with the SDQ total difficulties score. We found no significant links for enthusiasm.

**Table 6:** Multiple linear regression modeling for teachers' professional competence on teachers' SDQ ratings.

**Outcome**	**Predictor**	***b***	***b (95% CI)***	**ß**	***p^a^***	**Fit**
SDQ–total difficulties score	(Intercept)	18.9507	[5.79, 32.11]	−0.0132	0.867	
	Enthusiasm	0.5776	[−2.17, 3.32]	0.0409	0.678	
	Self-efficacy	−5.1857	[−8.3, −2.34]	−0.3491	<0.001^**^	
	Multicultural beliefs	0.0932	[−2.34, 1.01]	0.0080	0.924	
	Stereotypes	2.3846	[−1.84, 2.03]	0.2147	0.011^*^	
						Adj. *R^2^* = 0.1513^***^
Hyperactivity/inattention	(Intercept)	0.6541	[−0.37, 1.68]	−0.0139	0.859	
	Enthusiasm	0.0478	[−0.17, 0.26]	0.0433	0.660	
	Self-efficacy	−0.4318	[−0.65, −0.21]	−0.3729	<0.001^**^	
	Multicultural beliefs	0.1531	[0.00, 0.30]	0.1684	0.046	
	Stereotypes	0.2034	[0.06, 0.35]	0.2165	0.005^*^	
						Adj. *R^2^* = 0.1812^***^
Conduct problems	(Intercept)	0.7856	[−0.11, 1.68]	−0.0033	0.967	
	Enthusiasm	−0.0350	[−0.22, 0.15]	−0.0370	0.714	
	Self-efficacy	−0.1774	[−0.37, 0.02]	−0.1788	0.074	
	Multicultural beliefs	−0.0203	[−0.15, 0.11]	−0.0261	0.761	
	Stereotypes	0.1606	[0.04, 0.29]	0.2467	0.0112	
						Adj. *R^2^* = 0.0851^**^
Peer relationship problems	(Intercept)	1.3958	[0.56, 2.23]	−0.0072	0.930	
	Enthusiasm	0.0151	[−0.16, 0.19]	0.0173	0.865	
	Self-efficacy	−0.2329	[−0.41, −0.05]	−0.2550	0.012	
	Multicultural beliefs	−0.0667	[−0.19, 0.06]	−0.0931	0.284	
	Stereotypes	0.0733	[−0.04, 0.19]	0.1074	0.213	
						Adj. *R^2^* = 0.0738^**^
Emotional symptoms	(Intercept)	0.9869	[0.05, 1.93]	−0.0208	0.806	
	Enthusiasm	0.0861	[−0.11, 0.28]	0.0921	0.386	
	Self-efficacy	−0.1967	[−0.40, 0.01]	−0.2002	0.057	
	Multicultural beliefs	−0.0522	[−0.19, 0.09]	−0.0677	0.456	
	Stereotypes	0.0416	[−0.09, 0.17]	0.0566	0.527	
						Adj. *R^2^* = 0.0105
Prosocial behavior	(Intercept)	−0.2828	[−1.19, 0.63]	0.0279	0.718	
	Enthusiasm	0.1643	[−0.03, 0.36]	0.1649	0.091	
	Self-efficacy	0.3031	[0.11, 0.50]	0.2894	0.003^**^	
	Multicultural beliefs	0.0457	[−0.09, 0.18]	0.0557	0.499	
	Stereotypes	−0.1091	[−0.24, 0.02]	−0.1393	0.090	
						Adj. *R^2^* = 0.1998^***^

*b, unstandardized regression weights; b (95% CI), 95% confidence intervals for the unstandardized regression weights; ß, standardized regression weights; p, two-sided p-values; Adj. R^2^, adjusted R^2^*.

* *p < 0.05*,

** *p < 0.01*,

*** *p < 0.001*.

a *Markings indicating significances following one-sided Bonferroni–Holm correction. Prosocial behavior and total difficulties scores were not part of the hypothesis testing and Bonferroni-Holm adjustment was calculated separately*.

Regression analyses of demographic data on teachers' professional competence and perception of behavior problems revealed that teachers' work experience with newly arrived children was positively associated with both teachers' stereotypes, β = 0.218, *t*_(134)_ = 2.746, *p* = 0.021, and multicultural beliefs, β = 0.209, *t*_(134)_ = 2.749, *p* = 0.020. The other demographic teacher variables, work experience and school-leaving qualification, were neither associated with stereotypes nor multicultural beliefs. Furthermore, neither self-efficacy nor enthusiasm for teaching newly arrived children were significantly associated with any of the demographic teacher variables.

Teacher's work experience, β = −0.012, *t*_(134)_ = −2.575, *p* = 0.033, was negatively associated with the perception of total difficulties. Children's male gender was positively associated with the perception of total difficulties, β = 5.045, *t*_(114)_ = 3.958, *p* < 0.001. The perception of total difficulties was not associated with any other demographic child or teacher variable. For the SDQ subscales, children's male gender was positively associated with both perception of hyperactivity/inattention, β = 0.447, *t*_(114)_ = 4.461, *p* < 0.001, and the perception of conduct problems, β = 0.284, *t*_(114)_ = 3.424, *p* = 0.023. The children's male gender was also negatively associated with the perception of prosocial behavior, β = −0.352, *t*_(114)_ = −3.860, *p* = 0.001. Furthermore, children's age was significantly positively associated with the perception of prosocial behavior, β = 0.012, *t*_(114)_ = 3.217, *p* = 0.010. Considering the country of origin, children from the African continent (Egypt, Eritrea, Morocco, Nigeria) were rated higher on the conduct problems scale and above the average when compared to all children, β = 0.363, *t*_(114)_ = 3.337, *p* = 0.030. All other associations between teacher or child demographics and teacher's professional competence and perception of behavior problems were insignificant.

## Discussion

The aims of our study were two-fold: In the first part, we investigated the links between pre-school teachers' negative stereotypes, multicultural beliefs, self-efficacy, and enthusiasm when teaching newly arrived refugee children. In the second part, we investigated the links between those variables and teachers' perceptions of newly arrived refugee children's behavior problems. We found teachers' stereotypes and multicultural beliefs to be associated with their self-efficacy and enthusiasm for teaching refugee children. Moreover, we found that both teachers' stereotypes and self-efficacy were associated with their perceptions of externalizing behavior problems.

### First Hypothesis

Our first research aim was to investigate the links between teachers' stereotypes and multicultural beliefs toward refugee children as well as teachers' self-efficacy and enthusiasm for teaching refugee children. In line with previous research (19, 23–25), we found that negative associations between stereotypes and motivational orientations as well as positive associations between multicultural beliefs and motivational orientations hold for pre-school teachers teaching newly arrived refugee children.

In several studies, teachers have reported feeling insecure when working with refugee children (14–16). Teachers' self-efficacy has accordingly been linked to their well-being (47), suggesting that not only the well-being of refugee children but also the entire pre-school facility could benefit from considering the links between teachers' beliefs, values, and motivational orientations. Working on teachers' stereotypes and cultural beliefs may likely improve their self-efficacy and enthusiasm, which in turn could foster teachers' well-being and allow them to be more confident when teaching refugee children. As classroom quality is linked to child performance (48, 49), pre-schools with teachers displaying less stereotypes and agreeing higher with multicultural beliefs should be more effective in fostering academic and social-emotional learning in newly arrived refugee children. This is also likely to facilitate their transition into primary schools, as establishing a positive learning environment can be an important step toward catching up with learning delays and adapting to new and unfamiliar structures, e.g., during post-migration periods.

### Second Hypothesis

We found that teachers with more stereotypes were also more likely to perceive hyperactivity/inattention and total difficulties in newly arrived refugee children. This is in line with findings that teachers reported more behavior problems for certain subgroups of migrant or minority children (30, 31) and more attention problems in refugee children (6). Negative expectations toward refugee children could lead to a selective focus on more problematic aspects of child behaviors. However, our data support this notion only for externalizing behavior. This partially inconsistent pattern could be explained by the higher visibility of children's externalizing behavior problems over their internalizing problems. Surprisingly, teachers with more stereotypes did not perceive more conduct problems, which are also externalizing problems. Nonetheless, our evidence on this is at the borderline as the link between stereotypes and conduct problems had reached significance before Bonferroni-Holm correction (*p* = 0.012, one-sided). Future research should re-examine whether stereotypes are related to the perceptions of conduct problems as well.

We found no significant effects on behavior problems for multicultural beliefs. To the best of our knowledge, no prior evidence exists regarding the links between multicultural beliefs and perceptions. Possibly, multicultural beliefs reflect teachers' ideas on how refugee children could be integrated best–without impacting how they perceive refugee children's behaviors, emotions, or performance. Indeed, the items measuring multicultural beliefs did not refer to student achievements. During and after transitioning into primary school, however, self-regulation and navigation through social situations can become challenging for refugee children (17). Additionally, perceiving refugee children as more divergent can influence their school outcomes and performance (37). Thus, detecting conduct and other behavior problems as unbiased as possible is crucial for promoting refugee children's learning and positive development. Previous research found that teachers exhibited stereotypes toward refugee children (37). Teacher training aiming at improving early education for refugee children should thus focus on reducing teachers' stereotypes, whereas our findings suggest that considering the degree of multicultural beliefs is less important.

### Third Hypothesis

We found that teachers with higher self-efficacy were less likely to perceive hyperactivity/inattention and total difficulties in newly arrived refugee children. Consistently, previous research found that teachers' self-efficacy beliefs contributed to predicting their sense of efficacy in resolving cultural conflicts (32). Thus, teachers with higher self-efficacy might perceive certain child behaviors as less problematic, as they are more confident about their ability to solve potentially arising conflicts. In our investigation, perception bias could have also arisen when teachers with lower self-efficacy did not trust their abilities to detect behavior problems and thus reported lower scores in the SDQ subscales. Again, it is surprising that teachers with higher self-efficacy perceived less hyperactivity/inattention, but not less conduct problems. The items for identifying conduct problems refer to negative interactions with peers and adults. Items for hyperactivity/inattention do not differ between hyperactivity and inattention, while the latter might not be equally observable in German pre-school settings. In contrast to early education in other countries, pre-school curricula in Germany include more play-based learning. Hyperactivity/inattention symptoms might be less visible in play situations, as those are not extrinsically structured. This would allow for more interpretation and influence of stereotypes and self-efficacy on the teacher ratings.

We found no associations between teachers' enthusiasm and their perceptions of behavior problems. For the professional development of pre-school teachers, teachers' self-efficacy, rather than their enthusiasm, could be more important when preparing them for working with refugee children. However, teachers' enthusiasm was only assessed with two items and future research should consider expanding this measuring instrument. Interestingly, we also found that higher self-efficacy of teachers was associated with their perceptions of children's prosocial skills. We found no previous literature on this link. Future research is needed to clarify whether this association can be replicated.

### Perception of Externalizing Behavior Problems

We found hyperactivity/inattention to be associated with stereotypes and self-efficacy. This is in line with other evidence suggesting that teachers might be more sensitive to externalizing mental health problems than to internalizing mental health problems (6, 50). A possible reason could be that teachers' experiences are limited to pre-schools and that internalizing behavior might be more visible in home environments (51). In addition, children in our study were very young and at an age when externalizing behavior problems are more likely to be evident. Research suggests that young children tend to display externalizing behavior symptoms even when having internalizing disorders. For example, depressive symptoms were found to be manifest in externalizing and school problems in school-aged boys (52). As refugee children often show heightened mental health problems (4, 5), it is of special importance to raise teachers' awareness of possible underlying internalizing problems.

### Demographic Variables

In addition to our main analyses, we found that children's age, gender, and region of origin, as well as their teachers' work experience and work experience with refugee children were partly associated with teachers' professional competence and SDQ ratings. Teachers' work experience with refugee children was moreover related to the higher agreement with multicultural beliefs and more negative stereotypes. Such patterns suggest that working with refugee children influences teachers' stereotypes and cultural beliefs in a way that teachers who are more experienced in working with refugee children could view children's different backgrounds more positively and consider them when interacting with these children, but also are more likely to assume that refugee children encounter more difficulties in pre-school and are thus in need of more support from their teachers. Moreover, we found that teachers' work experience was negatively linked to the perception of total difficulties. Increasing work experience could thus be linked to less stigmatization of newly arrived refugee children as “traumatized,” a narrative that dominates public and partially also scientific discourses. Future research could clarify and further substantiate such associations.

Children's age was only positively associated with the perception of prosocial behavior, but not with SDQ problem scales. Possibly prosocial behavior is more visible in older children. Future research should re-investigate this possible link. Moreover, children's male gender was linked to increased perception of total difficulties and externalizing behavior problems (i.e., hyperactivity/inattention and conduct problems), as well as negatively associated with the perception of prosocial behavior. This is in line with previous research, in which teachers in day care centers perceived more externalizing behavior problems in boys (53). Also, teachers in elementary schools reported more hyperactivity/inattention for boys (54). We also found that newly arrived refugee children from Africa were reported to display more conduct problems. To the best of our knowledge, no previous literature has reported this link. Future research is needed to verify whether this finding can be replicated.

### Limitations and Future Directions

This study measured teachers' perceptions with questionnaires, but these data were not corroborated by other methods. To address this limitation, teachers were asked to rate the behavior problems of the child that had been in the pre-school facility for the longest time. With this approach, we hoped to reduce the bias of choosing the child with the most visible mental health symptoms. Since we were unable to verify whether teachers followed this guideline, we cannot exclude a selection process. Additionally, it remains unclear whether pre-school teachers' professional competence influences their perceptions of behavior problems, or whether refugee children's behavior problems in pre-schools shape teachers' professional competence. This should be considered in future research. Classroom determinants such as composition could help to clarify those relations and should be addressed in future research. Nonetheless, by only sampling one teacher and child per pre-school group, we ensured better generalizability across pre-schools. Teachers' own migrant background could be related to their professional competence and the way they perceive refugee children's behavior problems. This could be investigated in future research. We also assessed work experience with refugee children as an eligibility criterion. In our sample, pre-school teachers stated to be very experienced with refugee children, and they displayed rather high agreements with multicultural beliefs as well as self-efficacy and enthusiasm for working with newly arrived children. Thus, the generalizability of our results could be limited to pre-school teachers having already worked to some extent with newly arrived children. As findings from other studies suggest that teachers often lack experience or education in teaching refugee children (14–16), future research could examine whether our findings can be generalized to teachers with less work experience with refugee children.

## Conclusion

A large number of refugee children with heightened risks for behavior problems entered early education settings. Early education can decisively promote learning trajectories of pre-school-aged refugee children. The role of teachers' motivations, beliefs, and perceptions toward those children and their assessment abilities of behavior problems demand additional research. Teachers can provide important insights into the socio-emotional needs of young refugee children during their post-migration periods. Our findings suggest that teachers' stereotypes and self-efficacy might be related to perceptions of refugee children's externalizing behavior problems. Possible perception biases might thus be addressed by fostering teachers' professional competence and considering teachers' stereotypes and self-efficacy in teacher training. Furthermore, researchers should consider teachers' professional competence when interpreting mental health assessments of refugee children conducted by teachers.

## Data Availability Statement

The raw data supporting the conclusions of this article will be made available by the authors, without undue reservation.

## Ethics Statement

The studies involving human participants were reviewed and approved by the Ethics Committee of the Faculty of Psychology of the Ruhr-University Bochum. The participants provided their written informed consent to participate in this study.

## Author Contributions

SC contributed to manuscript writing and revision, study conceptualization, data analysis, and interpretation. BL contributed to study conceptualization, funding acquisition, manuscript feedback, and revision. AH contributed to study conceptualization and implementation. CB contributed to data curation and data analysis. JB contributed to study conceptualization, data analysis and interpretation, supervision of the research project, manuscript feedback, and revision. All authors contributed to the article and approved the submitted version.

## Conflict of Interest

The authors declare that the research was conducted in the absence of any commercial or financial relationships that could be construed as a potential conflict of interest.
